# The causal configurations of provincial health policy innovation in China: an analysis of the food safety standard filing policy

**DOI:** 10.3389/fpubh.2023.1259717

**Published:** 2023-11-30

**Authors:** Li Li, Guanghua Han, Yanting Chen, Zilin Zhang, Xiao Fu

**Affiliations:** ^1^Institute of Healthy Yangtze River Delta, School of International and Public Affairs, Shanghai Jiao Tong University, Shanghai, China; ^2^Lord Byng Secondary School, Vancouver, BC, Canada; ^3^Zhejiang Informatization Development Institute, Hangzhou Dianzi University, Hangzhou, Zhejiang, China

**Keywords:** policy innovation, food safety, enterprise standards, filing system, qualitative comparison

## Abstract

**Introduction:**

According to China’s Food Safety Law of 2015, the filing of food safety enterprise standards is a policy innovation led by p9rovincial governments in China. However, there are significant differences in the development of the “Food Safety Enterprise Standard Filing Policy” between provincial governments across the country. This study aims to explore the internal mechanisms driving autonomous innovation by provincial governments in the absence of administrative pressure from the central government, to better understand the policy innovation mechanism in the Chinese context.

**Methods:**

Crispy Set Qualitative Comparative Analysis (csQCA) method is used to identify the innovation mechanism.

**Results:**

This study found that provinces with good provincial economic resources and strong government capabilities are prone to policy innovation, and the influence of internal factors of provincial governments is stronger than that of external factors.

**Discussion:**

When provincial economic resources and capacity are weak, endogenous factors in the province also help achieve proactive policy innovation by provincial governments. The research results reveal how provincial governments construct local policies in the absence of administrative pressure from the central government.

## 1 Introduction

Food safety has long been an important issue of widespread concern in China society. Enterprise standards are tools for food production enterprises to guide production, process management, food safety, and other tasks to ensure food safety (1). Therefore, enterprise standards have been formulated by China’s central government. After the implementation of China’s Food Safety Law 2015, the standard preparation system for food safety enterprises changed, and the central government handed over the right to formulate the system to provincial health departments. Accordingly, provincial health departments implement the filing system on their own under the comprehensive lead of provincial governments. The change in policy preparation for food safety enterprises is an innovation of the government administration, which is conducive to enterprises paying attention to food health and safety and putting forward their own food safety standards in accordance with food safety requirements.

As a factor conditioning production and inspection in food businesses, food standards influence the health of the population (2). Food companies in China are required to produce products that meet the national quality standards. Currently, the health administrations of most provinces in China have completed procedures for filing food safety standards with food safety companies. Without the involvement of the central government, provinces are in the process of developing or amending local food safety standards filing systems. However, the development of this policy has progresses to different extents in different areas. From this perspective, there is a pressing need to identify the factors that influence the proliferation of provincial innovation and how they interact to create innovation in provincial government policies.

Policy innovation is understood as the creation or introduction of solutions to resolve problems, overcome challenges, or seize opportunities in political systems (3). While policy innovation is a more traditional research topic, decentralization reforms, in which the central government devolves economic and administrative authority to provincial governments, have been undertaken in recent years in a large part of the world (4), whereby provincial governments undertake policy innovations to facilitate governance transformation. Differences in political systems and cultural traditions mean that provincial governments in different nation-states form unique paths of policy innovation, resulting in enormous differences in their patterns of behavior and outcomes. Therefore, provincial governments in both democracies and authoritarian countries have an intrinsic drive to stimulate policy innovations, and their innovative reproduction has become one of the most important practices in countries around the world (5).

China’s political system is characterized by a socialism with China top-down managerial characteristics, which leads provincial government officials to behave differently from their Western counterparts. Within this political structure, provincial officials select development strategies to their own advantage based on the available resources. China’s local bureaucratic system is characterized by fiscal half-power, administrative half-independent, with the central government playing an important role in driving innovation in provincial governments (6). Given that much of the literature on innovation in provincial governments has been published in Western democracies (7), provincial government innovation should spread across countries to enrich the logic of policy innovation in provincial governments. However, the logic of provincial innovation has been ignored in many studies in China, with only a few case studies examining it (8–10). The Food Safety Enterprise Standard Filling Policy (FSes) is a health management policy that has been in place in China for 8 years, under which the central government has authorized provincial governments to develop provincial policies. FSes policy thus affords researchers the opportunity to examine provincial innovation in the Chinese context and further test the applicability of the policy proliferation theory in China.

## 2 Theoretical framework

Policy innovations can help identify and accumulate knowledge about the determinants of policy uptake, and numerous studies on the proliferation of policies have been published over recent decades (11) (10). The study of policy diffusion is of great theoretical and practical importance as government policies have been documented to be transmitted through either vertical or horizontal mechanisms (12–15). However, the factors influencing policy innovation are multifaceted, including both horizontal and vertical ones. Horizontal proliferation occurs between neighboring governments, whereby new policies are built through learning, mimicry, and competition. When the geographic proximity of the intergovernmental development environment at the same level is similar, the communication of information is more convenient, provides favorable conditions for horizontal policy diffusion, is more applicable to policy replication in neighboring provinces, and reduces the potential for trial and error. Vertical diffusion reflects the vertical relationship between two levels of government, with empirical replication at the bottom and mandates at the top.

In addition to horizontal and vertical diffusion models, internal and external models have been widely used to explain the mechanisms of policy innovation (16). It has become conventional to explain policy innovations as an interdependent policy making process shaped by both internal and external factors. Normally, the internal factors represent the capacity of the government itself (e.g., organizational structure and resource capacity), as well as factors related to jurisdiction (covering local social, political, economic, cultural, and natural aspects). External factors are primarily the aforementioned pattern of vertical and horizontal proliferation and domestic and foreign political, economic, environmental, and other factors.

The fact that China is very different from typical Western countries in terms of economic development, political uniqueness, and Confucianism makes it an excellent case study of the universality of political proliferation theory. In the context of China, the internal-external mechanism is very helpful to explain influencing factors for China’s political innovations. Traditionally, China has been considered to be run by top-down structured governmental systems (6), and the activities of governmental policy innovations significantly depend on the non-western bureaucratic system. In China, main provincial government leaders have possibility to be promoted by the central government. Because neighboring provinces have many economic and demographic similarities, provincial government leaders may view peers in neighborhoods province as competitors (17). As a result, provincial political innovations partly depend on the political innovations of competitive provinces. Competition between neighboring provinces is a key external determinant of provincial political innovation.

Meanwhile, the internal factors of provincial innovation in China are more diverse. As a developing country with the world’s largest population, China has measured government performance primarily by economic outcomes since ethe 1980s (18). Thus, the government has given strong incentives to support the companies under their regime and respond to companies’ requirements for local policies. The abilities of provincial governments to enact political innovation include the economic ability to apply innovation, the professional ability to conceive local polices, and the leadership ability to develop innovation programs. Therefore, we consider government capacity, socioeconomic development, and professional authorities as internal factors for provincial policy innovations.

### 2.1 Government capacity

China’s reform and opening-up process was initiated in 1978, aimed at unlocking the nation’s growth potential through a dynamic socialist market economy (19). In addition to the development of the concept of socialism, the central government gradually reformed the highly centralized management model inherited from the socialist planned economy. The central government accepted the need to devolve its economic power to the provincial governments, encourage policies that foster institutional innovation, and avoid the need for reformist leaps and bounds (20–22). Following improvements in the tax system in 1994, the central and provincial governments formed a shared tax distribution structure, and the central government secured the vast majority of fiscal resources for redistributing fiscal transfers, balancing regional economic differences among provincial governments. Relative autonomy has been granted to provincial governments to initiate innovative projects and build local financial capacities (23). As a result, provincial governments have a greater incentive to adopt innovative policies and measures to develop the local economy, raise revenue, and reduce financial expenditure according to the actual situation of the local economy.

When government finances run large surpluses, it is best to stimulate government innovation, which in turn drives government innovation policies (24). Studies have shown a clear positive correlation between provincial government revenue and provincial political innovations in China (25). Therefore, the actual implementation of policies depends on the level of government input. Without substantial financial support, provincial governments have little incentive to implement such projects. Simultaneously, the theory of resource relaxation posits that innovation is more likely to occur under conditions of excess organizational resources. In recent years, with the slowing economic growth of our country, provincial governments have faced unprecedented financial pressure. Even developed provinces and cities such as Beijing and Guangdong face funding shortfalls as revenue growth slows sharply. However, when the attention, time, and resources of lower levels of government are limited, the level of policy innovation varies from province to province.

The rule of law is one measure of the modernization of governance (26). Under strong rule of law, governments ensure quality, stability, and access to justice for all members of society. Thus, rule of law in good governance indicates that a legal framework is in place that empowers the government in governance (27). With a good legal system, provincial governments have accumulated a great deal of experience in building a system of rule of law, which is useful for tracking policy changes quickly at higher levels. On the other hand, strong experience in provincial government provides reformers with greater psychological security in that they can make mistakes or take risks, thus increasing the likelihood of another reform (28). Standard filing system for food safety companies is itself a legal system with the provincial government as the main body, as well as a system for managing businesses in the area. Food safety is a common concern in society that affects the health of all consumers (29), legal system for the regulation of food safety is highly technical and demanding. As a result, the greater the degree of rule-of-law, the more provincial governments enable to formulate laws and systems consistent with the current legal system to avoid food-safety-related loopholes.

### 2.2 Socioeconomic developments

Socioeconomic developments include the economic level of provinces, economic growth, industrial structure, and the number of foreign-owned enterprises (30–33) are also considered in this study. An example of the impact of socioeconomic development on policy innovation can be seen in the evaluation of provincial government leaders in China. Given that economic performance is a primary criterion for assessing and promoting local leaders in the past several decades in China, provincial governments have a strong incentive to support economic growth through market-driven innovation. Filing systems provide a legal basis for food safety companies to engage in their production and operations. The early establishment of a local legal system at the behest of a higher government can provide the corresponding direction and adaptation to the needs of society and ultimately foster the development of the local economy.

On the other hand, socioeconomic factors may have a “pressure valve effect” on innovation in provincial government, meaning that when an area is more economically active there is a lobbying presence. Lobbying involves efforts to deliver pressure on policy makers to secure desired political outcomes (34). In Western countries, interest groups lobby provincial governments for economic benefits to facilitate the passage of legislation and institutions that benefit them (35–37). Although there is no lobbying in China, companies work more closely with provincial governments. If the business is not only a major provincial tax payer but also the primary agency to absorb local jobs, food production, and operations companies, the provincial government should establish a closer symbiotic relationship. When the economy is active, firms work more closely and harmoniously with provincial governments. Thus, when the economy is active, provincial governments have incentives to engage in institutional innovation based on their business needs. As the main body for adjusting the standard filing policy of food safety firms, the amount and size of the food industry have an important influence on the requirements for standard filing policies and also constitute a source of pressure when supervisory authorities oversee them. As the number of food producers increases, so does the number of business activities (design, production, and technical marketing) that require provincial government regulation as a guide to facilitate governmental structures (38, 39).

### 2.3 Professional authorities in leadership

The capacity of provincial government to innovate as an agent of policy innovation is a product of organizational functioning (40). Decentralization between government departments, however, also makes cross-sectoral cooperation challenging and detrimental to the governance of social issues (41). Schick (42) argues that successful budgeting for government performance requires strong leadership; otherwise, the process will face a great deal of resistance and obstacles. Throughout the 1980s and 1990s, the US federal government took steps to decentralize the control of certain policy areas such as social assistance to the states and localities of the country. More than a dozen states do not employ a large number of lawmakers and legislatures meet for no more than a few months each year.

Consequently, legislators tend to address the most urgent tasks, and autonomous and innovative policies are not on the legislative agenda (35). This is one of the main reasons legislators must deal with the most pressing issues. China has launched a model of policy innovation and diffusion in the field of river pollution control in the name of “river chief.” China’s waterways have been polluted for years, and the economic effects of the rivers that flow through the provinces have been marred by their commercial activities. Cooperative governance between provinces has always been an issue since provincial governments are reluctant to cooperate out of concern for the economic benefits of their local areas. China has adopted a multisectoral “river chief” policy to control river pollution, and a “river chief” should be appointed to oversee the protection of every river in the country. Therefore, each province has applied the “river chief” policy within its administration region since 2016. River chiefs are authorized to lead river protections against pollution, and the institution has been an effective policy for solving complex collaborative problems in river management. The water quality in China has improved significantly in the past several years because of the “river chief” policy, which indicates that organizations with authorized power contribute strongly to innovation in government management.

Under the Chinese bureaucratic system, the responsibility of provincial health departments to manage the standard filing policies of food safety enterprises is broken down into internal agencies that are specifically responsible for the standard filing of food safety enterprises. The internal agency must complete a series of studies and prepare the food safety company’s standard filing policy, then report it to the provincial health department and, after consideration by the meeting, to the provincial legal department. The innovation agency’s policy function is to exercise the power of its suggestions during this process. Reform efforts in China often require cooperation and coordination among multiple stakeholders from different sectors (such as audits, inspections, and personnel), comprehensive knowledge, prior experience with reform, and leadership in relevant professions (28). Thus, independent innovation agencies are professionalized and empowered institutions in the field of policy innovation, facilitating multi-stakeholder cooperation and policy innovation.

### 2.4 Neighborhood learning and competition

Horizontal diffusion is often driven by learning, imitation, and competition, whereby the government attempts to reduce the cost of trial and error and improve performance by mimicking other governmental practices. It has similar political and economic structures when combined in geographic clusters. In many cases, governments draw advice and lessons from other provinces, especially those with similar economic or political standing, which helps reduce the costs and risks that may be absorbed into the decision-making process (43). Through policy innovations between neighboring states, for example, the United States has shown that geographic proximity between governments improves the efficiency of information diffusion and facilitates the adoption of new policies by policy adopters.

China’s central government has since devolved significant economic-related administrative functions and powers to the provincial government (44–45), which has been the focus of much of this research. Under China’s political system, the central government enable to limit the direction or scope of reforms through the direct issuance of policy documents or by appointing and removing provincial government leaders via performance reviews (46). Provincial governments may view peers in similar economic situations as competitors, with limited funding or political support from higher governments. If certain policies are adopted by one province, other provinces with similar economic structures may be under competitive pressure to adopt the same policies in order to avoid becoming overwhelmed. Consequently, with most neighboring provincial governments adopting a food safety grading system, it is easy for provincial governments to adopt this innovation.

## 3 Method and data analysis

The political, economic, and social environments of different provinces vary, and the formulation of public policies in each province is unique. The uniqueness of the individual cases coincides with the coherent analytical idea of qualitative comparative analysis. This retrospective case review allowed the researchers to interpret specific cases as much as possible and gain inspiration. This article, based on the chronology of the review of the provisions of the Food Safety Law of the Twelfth National People’s Congress Standing Committee on April 24, 2015, on the lodging of food safety standards for food safety companies, investigates the sequence of measures canceling and revising the filing system for food production firms in each province as measures of the diffusion of policy innovation, taking provinces as the basic unit of research and combining the cancelation and revision of the food safety company filing system in the 31 provinces (including provinces, autonomous regions, and municipalities).

Based on the explanation and statistics noted above, the newly revised provincial food safety enterprise standard filing systems implemented by the 31 provincial health administrative departments was used as the outcome variable in this study. Based on provincial health administration statistics on the timing of the implementation of the newly revised standard food safety business filing system, the implementation timing reflects provincial government uptake and perception of the policy and whether there is innovative policy proliferation. Given that the event observation endpoint for policy innovation in this study was the formal publication of normative documents (the abolition or a new filing system of food safety standards for enterprises), the production and filing of normative documents should be strictly in accordance with the degree of provincial rule-of-law, with a typical cycle of 60 days. When we assigned the time nodes in Table 1, we treated dates that differed by no more than 60 natural days as juxtaposed data.

**Table 1:** Values of explanatory variables.

CASEID	PFA	GDP	DOR	FISV	INP	IA
AH	2454.3	35,997	199.56	661.64	2/3	1
BJ	4723.86	106,497	224.18	281.77	1/2	1
FJ	2544.24	67,966	197.06	1258.45	1	0
GS	743.86	26,165	199.09	77.9	5/6	0
GD	9366.78	67,503	234.43	1751.52	1/5	1
GX	1515.16	35,190	198.18	363.58	1/5	0
GZ	1503.38	29,847	214.66	157.05	3/5	0
HAN	627.7	40,818	180.02	43.71	1	0
HEB	2649.18	40,255	171.18	1022.72	5/7	1
HEN	3016.05	39,123	194.18	2803.26	1/6	0
HLJ	1165.88	39,462	214.83	649.73	1	1
HUB	3005.53	50,654	184.98	1172.91	3/7	1
HUN	2515.43	42,752	207.87	1020.43	1	0
JL	1229.35	51,086	184.49	460.3	2/3	1
JS	8028.59	87,995	214.17	946.01	1/4	1
JX	2165.74	36,724	222.75	536.63	0	0
LN	2127.39	65,354	186.62	441.95	0	0
NMG	1964.48	71,101	171.1	680.96	1/2	0
NX	373.45	43,805	154.99	154.76	0	0
QH	267.13	41,252	172.23	32.68	0	1
SD	5529.33	64,168	202.56	2637.18	1	0
SX	1642.35	34,919	157.3	117.97	0	0
SAX	2059.95	47,626	168.5	487.94	1	0
SH	5519.5	103,796	230.44	598.65	1	1
SC	3355.44	36,755	221.14	971.35	3/7	1
TJ	2667.11	107,960	180.7	1347.53	0	1
XZ	137.13	31,999	125.76	5.92	1/2	0
XJ	1330.85	40,036	155.65	218.92	1/3	0
YN	1808.15	28,806	190.83	201.29	1	1
ZJ	4809.94	77,644	214.13	534.74	1/5	1
CQ	2154.83	52,321	212.19	232.13	0	1

Government capacity for food policy innovations includes the financial ability to stimulate innovations and the professional ability to create political innovations (47). Following Nan et al. (48), we measure provincial financial ability (PFA) from the China Statistical Yearbook (2016). The legal system for the innovation of food safety policy is highly technical and professional. As a result, the greater the degree of rule of law, the more provincial governments will be able to formulate legal and policy systems consistent with the current legal system. Following Cai and Wang (49), we measure the provincial degree of rule-of-law (DOR) based on data from Annual Assessment Report on China’s Law-Abiding Government (50).

Since GDP (Gross Domestic Product) is a key indicator of market-related economic activities in a country, we use provincial GDP *per capita* to estimate provincial socioeconomic developments (51). Since business activities (design, production, and technical marketing) require provincial government regulation as a guide (38, 39), the food producers constitute a source of pressure upon government for impartial legal innovation of regulations. The business activities of food industries can be indicated by Food Industry Sales Value (FISV). We obtain the census data for FISV from the China Food Industry Yearbook (52). According to the Regulation of the People’s Republic of China on the Disclosure of Government Information (53), the agencies of provincial governments are required to publicly disclose all bureau institutions. Thus, we counted the provinces that have built an independent agency (IA) for the management of the standard filing policy for food safety before 2015 on the website of each provincial government. We calculated the number of provinces that were the first to implement the policy as a percentage of the provinces that were adjacent to it; the order of time points at which provinces implemented the policy is examined. Following Singu (54), we measure INP (influence of neighboring provinces) by examining whether a policy innovation occurs in neighboring provinces before the time that a province proposes its own policy innovation. The values of the six variables chosen based on the research framework given above are illustrated in Table 1.

Variables are dichotomized according to the basic principles of partitioning, which makes the distribution of examples more meaningful and easier to align with existing theories, thereby supporting the correlation between variables and positive results. Results: When the variable was binomial, the optimal ratio of no cases (0 assignments) to cases (1 assignment) was 1:3. Therefore, we assigned zero values to 1 of the 23 positive cases and 1 of the 8 negative cases. This study concludes that the distribution of these two variables is the same across provinces, both in terms of the time taken to repeal the policy and the time it was revised. Therefore, for ease of description, the time at which the policy was repealed was selected as the outcome variable.

The time point at which the variables were observed was November 4, 2016; therefore, we classified them according to the time point of November 4, 2016. The province ahead of the time node was considered to have an administrative pressure value of 0 at the upper level or 1 at the lower level. The establishment of a separate office with organizational characteristics caused us to assign the province a value of 1; if there was no separate office or organizational functions, it was assigned 0. Because of the large variances and variances in other precursor variables, the median rather than the mean was used as a mechanical cutoff point to spread the cases more evenly.

In all, 31 samples were selected as case analysis units from all provinces of mainland China. The Crispy Set Qualitative Comparative Analysis (csQCA) methods is used to explore the causal configurations in this study. The first step was to select cases supported by the theoretical identification of predictors and outcome variables. In the second step, each variable was dichotomized by fact and theory and described in a binary language. The third step was to set up the truth table, perform a standardized analysis, obtain the preset configuration, and impose the single-element or multi-element joint detection requirement according to the specific situation. In the fourth step, the combination path of each element was summarized and analyzed, and the discovery and revelation of each element were reviewed. On this basis, a binary distribution was created between one outcome variable and seven predictive variables, resulting in factual values for the 31 provincial cases (Appendix Table A1). To maximize the combination of key sufficient conditions and ensure that each antecedent variable is an indispensable and sufficient condition for the outcome, it is necessary to first analyze the necessity of each variable and then construct a table of true values and standardize the analysis (Table 2).

**Table 2:** Necessity analysis of explanatory variables.

Explanatory variables	Consistency	Coverage rate
PFA	0.608696	0.875000
GDP	0.608696	0.875000
DOR	0.478261	0.687500
FISV	0.608696	0.875000
INP	0.434783	0.625000
IA	0.565217	0.866667
APAP	1.000000	1.000000

Outcome variable: APAP.

In all, 64 conditional combinations were obtained using two classification assignments based on the six interpretive variables. Path selection was based on the principle that case frequency must be no less than 1 and consistency must be no less than 0.8, yielding the following table of truth values. Subsequently, a table of truth values was provided to determine the solution of the path. In this study, we selected the three combination paths with the highest coverage (0.261, 0.217, and 0.174).

Path A1 = PFA × GDP × DOR × IA.

Path A2 = PFA × GDP × FISV × ~INP × IA.

Path B = ~PFA × ~DOR × ~FISV × ~INP × ~IA.

Variables that appear in concise and intermediate solutions are generally considered core factors (Appendix Tables A2–A4), whereas variables that do not appear in concise solutions are considered secondary factors. The path configurations are listed in Table 3.

**Table 3:** Configurations of paths.

	Path A1	Path A2	Path B
PFA	●	●	⊗
GDP	●	●	
DOR	●		⊗
FISV		●	⊗
INP			⊗
IA	●	●	⊗
Coverage	0.261	0.217	0.174
Unique coverage	0.13	0.087	0.087
Cases	Beijing, Shanghai, Jiangshu, Zhejiang, Guangdong, Chongqing	Hubei, Tianjin, Guangdong, Jiangsu, Zhejiang	Ningxia, Shanxi, Liaoning, Xinjiang
Total consistency	1		
Total coverage	0.87		

The overall path consistency in Table 3 is 1, indicating a high correlation between the configuration combinations and results. The total coverage was 0.87 or greater, suggesting that the configuration may provide a more robust interpretation of the results. Path A1 had a coverage of 0.261 and a net coverage of 0.13 among the three paths chosen, while the Path A2 coverage was 0.217 and the Path B2 coverage was 0.174. The results for the A1 and A2 core conditions of the GDP agree with the IA. Simultaneously, A1 and A2 had similar configurations, with representative instances having overlapping instances.

Three pathways cover 12 cases, or 39 per cent of the 31 cases. Paths A1 and A2 comprise Jiangsu, Zhejiang, and Guangdong Provinces, all of which are southeastern coastal provinces. Some Path A1 cases are Beijing, Shanghai, and Chongqing, all of which are municipalities directly under the Central Government of China, with better provincial economic resources and more rapid economic development. The cases in the Path A2 group include Hubei and Tianjin. These areas have good geographical locations, well-developed transport systems, and large food industries. Route B includes four provinces, Ningxia, Xinjiang, Liaoning, and Shanxi, which are located in the northern part of China, are relatively remote, and have a low degree of governmental rule-of-law.

These three paths contain four core conditions: GDP, IA, DOR, and INP. IA reflects the distribution of administrative resources in the organizational structure of the health sector in the provinces, DOR reflects the local legal system, and INP reflects the influence of neighboring provinces. Based on these four core conditions and cases, the path of provincial government policy innovation can be divided into the following two categories:

First, the path is driven by provincial economic resources and government capacity. It contains two sub-pathways, A1 and A2, with GDP and IA as its core factors. The cases received through this path were mainly from provinces and municipalities with developed economic and geographical advantages. At the same time, these provinces have better local legal systems, mature markets (e.g., Shanghai and Jiangsu), and strong government capacity (e.g., Beijing and Tianjin). In other words, they have advantages in terms of both fiscal capacity and responsiveness to the government, which makes them highly susceptible to policy innovations.

The second is the endogenous innovation path, or the B-configuration path. This path had two core factors: DOR and INP. As a result, the corresponding regions are not highly regulated by law, and dissemination policies at the level of neighboring provinces result in less inter-provincial competition and relatively remote geographical locations (e.g., Liaoning and Xinjiang). These findings suggest that when a province faces a vertical or horizontal combination of high administrative pressure and a high degree of government rule-of-law, it may be difficult for it to break through existing legal frameworks and achieve policy innovation during periods of ambiguity. In contrast, geographically isolated and less rule-of-law provinces can be protected from excessive external pressures, and policy innovation and contagion can be promoted.

## 4 Discussions and political insights

Provincial government discretion in particular policy areas is a key condition that underpins the existing research on policy innovation. Thus, innovation in provincial government policy is not confined to typical Western countries. This type of policy phenomenon exists in developing, nondemocratic, and authoritarian countries, where policy innovation by provincial governments can be achieved as long as provincial governments have ownership over policy formulation. However, in an economical developing socialist country, such as China, provincial government policy innovation has unique characteristics.

In China, the main political leaders, such as secretaries of party committees, of provincial governments are generally not elected by the citizens of the provinces under their jurisdiction with one person one vote. Rather, appointments of secretaries of party committees are chosen by the Communist Party’s parent committee on the basis of economic performance, social stability, or political affiliation (55, 56). Local leaders are given carte blanche by the central government to determine their political careers, including evaluation, supervision, appointment, promotion, rotation, and demotion (57). Central policy signals must be given high importance by provincial governments. If the central government advocates a specific policy, it may be adopted by the provincial government in order to receive praise or attention from the central government. What are the factors that influence provincial government’s attempts to innovate when they are not required by the central government to take a particular action? This is the focus of this study.

Based on the theory of policy innovation, this study selected five influencing factors: government fiscal capacity, provincial economic resources, policy needs, neighborhood influence, and organizational characteristics. Subsequently, a policy innovation impact model framework was built based on these influential factors. Second, beginning with the adjustment of the standard food safety company filing policy following the Food Safety Law 2015, the qualitative comparative analysis method of the clear set was used to discuss the combination of factors affecting the standard deposit policy of food safety firms (58). Following the dichotomy of assignment, necessity testing, truth table construction, and norm analysis, three types of pathways for promoting policy innovation were analyzed. In this study, we integrated various horizontal and vertical proliferation mechanisms and tested them based on the proliferation of provincial government policies. The results of our research show that it is of great theoretical and practical importance to select appropriate methods for determining the specific mechanism of the policy proliferation process outside the Western context.

### 4.1 Economically developed provinces with sufficient resource and governmental capacity to have healthy policy innovations

The two basic conditions in the first path, GDP and IA, represent good provincial economic resources and a good organizational structure. Combined with GDP and IA, this can have a significant positive impact on policy innovation outcomes. Moreover, the greater the government revenue, the greater the impact. Theoretically, to the extent that lower-level governments are politically or financially constrained by higher-level governments, the former may be responsive to the latter’s policy preferences. The resource relaxation hypothesis suggests that policy innovation and diffusion activities are more likely to occur when the levels of government capacity are excessive relative to government resources (15, 59). Limited by the attention, time, and resources of lower-level governments, provinces with better resource endowments can respond more quickly to the demands of higher-level governments.

Turning to the mechanism of public policy innovation, there is a need to analyze China’s institutional context. Unlike Western democratic elections, China has its own bureaucratic system (60). Provincial officers work around the Western electoral system to win the support of voters and interest groups (61). Competition among provinces is strongly linked to elections. Under the Chinese bureaucratic system, the provincial government is more concerned with its performance and promotion, which are controlled by the central government. Innovation at the provincial government level demonstrates that provincial government leaders are loyal to the central government and are responsive to change, while economically developed provinces are more concerned about being perceived as inefficient. Regions with better resource endowments, such as Beijing and Shanghai, have higher levels of motivation and ability to respond to central policies in a timely manner. Beijing, for example, as China’s capital and political hub, has a strong government capacity and is easier to recognize and promulgate policies. Shanghai is located at the leading edge of China’s Yangtze River Delta region, where the economy is highly developed, transportation is convenient, and resources are relatively concentrated. In this respect, these two provinces had among the most innovative policies.

### 4.2 Less economically developed province with low legalized government and provincial competition are core determinants for political innovation

The empirical results of this study suggest that DOR and INP are fundamental determinants of endogenous innovation pathways, each of which represents a low level of regional case law and are less influenced by neighboring competition. The provinces of Xinjiang, Liaoning, and Ningxia have low levels of legalized governments and little interprovincial competition. All these provinces share the same characteristics, including being economically underdeveloped, relatively geographically remote, and having few policy innovations in neighboring provinces. In other words, the level of the legal system is not high, the lack of provincial competition in the less-developed sectors of the economy is the same, and political innovation occurs easily. Finally, in Section II, we mention that the levels of rule-of-law, competition from provinces, and economic development have positive impacts on political innovation. However, in the absence of these three fundamental factors, provincial governments can innovate more rapidly.

The degree of government rule-of-law is a central factor affecting the capacity of provincial governments to place greater emphasis on the local rule of law (62). However, this study finds that the degree of rule-of-law of a provincial government is not the core factor in its innovation measures. Conversely, provinces with low government rule-of-law are prone to policy innovation. This finding shows that there is a complex relationship between the level of rule-of-law of provincial governments and political innovation. The government, with its high degree of rule-of-law, strictly executes social governance according to the rule of law. The innovation of local policy involves reforming the existing system and improving the existing rules. The highly legalized government sets an example for government employees to enforce the law, which is not conducive to creating an administrative culture that improves existing laws and policies. Meanwhile, innovation in provincial government policies must be consistent with existing legal arrangements. Therefore, provincial governments with higher levels of rule-of-law are more willing to comply with the law. Where policy innovations conflict with existing legal arrangements, they must be pursued in conjunction with the relevant legislative branches. Provincial governments with higher levels of rule-of-law thus face greater challenges regarding policy innovation.

Geographical proximity is a frequently cited factor in innovative mechanisms (35). Many policy innovations confirm that geographical proximity promotes policy learning and that competition is a driver of mutual learning in adjacent regions (63). Where policy innovations do not occur in neighboring provinces, provincial governments typically lack learning opportunities and motivation, but when there is no policy innovation in neighboring provinces, it also affords provinces an opportunity to demonstrate the results of policy innovation under the policy and to be more easily recognized and supported by the central government (64). Although economic underdevelopment can hinder the economic basis of innovation, provincial governments in China have the opportunity to innovate through a cross-regional policy of peer support programs. In the 1970s, China began a policy of reciprocal support for ethnic minority areas and border regions. China supports Xinjiang and other locations through project assistance and the transfer of human resources, which also allows for the diffusion of new governance ideas and experiences in remote areas. For example, experience suggests that intergovernmental exchange fosters policy innovation in assisted areas (65). Counterpart aid has provided significant assistance to provincial governments in the remote parts of China in achieving policy innovation.

### 4.3 Internal factors play a more positive role in policy innovation than that of external factors

In the necessity test, we found the highest consistency and coverage of three variables: PFA (Local Public Finance Revenue), GDP (GDP *per capita*), and FISV (Food Industry Sales Value). Therefore, this study concludes that internal factors are more likely to lead to policy innovation than external factors. This finding can be explained in terms of China’s political system.

In recent decades, decentralization reforms have largely been implemented between central and provincial governments (66). Although China’s central government can still control the political mobility of provincial leaders through a crony-class personnel system, “Chinese federalism” (67) provides provincial governments with sufficient policy autonomy and protects their jurisdictions from political interference by the central government. Provincial governments support economic growth and social development by maintaining policy autonomy and formulating policies that are locally appropriate (68, 69). For provincial governments, enacting policies also has the important benefit of attracting the attention of the central government and ultimately indirectly preserving provincial autonomy by demonstrating their capacity.

Moreover, we found that IAs have a significant positive effect on policy innovation. Institutions with complex internal organizational structures find it difficult to coordinate with stakeholders, making policy innovation difficult (71, 72). According to China’s hierarchical governance rules, named the “three fixes” (70), independent agencies have authorized responsibilities and assigned resources and are empowered to perform their functions independently. Therefore, provincial governments that establish agencies with independent powers often break down organizational complexities, resulting in rapid professional innovation. The independent Food Safety Administration Office was established as the agency for this study and completed the strategic innovation of standard filing by food safety firms relatively quickly. This means that independent internal agencies with more defined roles are more focused on transforming new policies and may be supplemented by more administrative resources to absorb and transform relevant policies.

## 5 Conclusion

Over the past few decades, many countries have joined the global decentralization trend. Various economic and administrative powers have been devolved to provincial governments, many of which decide their own local policies on a case-by-case basis. Innovation in provincial government policies has become a popular topic. However, research on the diffusion of policy innovation in classical theory is generally based on a decentralized democracy. With sufficient discretion, provincial governments in non-democratic countries can also derive innovations.

Thus, in a socialist country such as China, the path of policy innovation by provincial governments is a meaningful research topic. We expect to conduct more research on policy innovation in various policy contexts. Provincial government innovation in China is not new, but has been a common phenomenon in China’s provincial government policymaking process for many years. Prior studies have typically examined the existence of policy proliferation and its influencing factors but have rarely shown the means of achieving policy innovation. In this regard, this study incorporates a variety of horizontal proliferation mechanisms to test health policy diffusion by provincial governments in China. The cases considered in this study provide a typical example of the mechanism for the diffusion of policy innovation by provincial governments in China without guidance from the central government.

## Data availability statement

The original contributions presented in the study are included in the article/Supplementary material, further inquiries can be directed to the corresponding author.

## Author contributions

LL: Formal analysis, Methodology, Project administration, Resources, Validation, Visualization, Writing – original draft. GH: Conceptualization, Investigation, Software, Supervision, Writing – original draft, Writing – review & editing. YC: Validation, Visualization, Writing – original draft, Writing – review & editing. ZZ: Data curation, Formal analysis, Investigation, Methodology, Software, Visualization, Writing – original draft. XF: Investigation, Funding acquisition, Formal analysis, Writing – original draft, Writing – review & editing.
