# Supplementary material for: The causal configurations of provincial health policy innovation in China: an analysis of the food safety standard filing policy
Source: Front Public Health. 2023 Nov 30;11:1259717. doi: 10.3389/fpubh.2023.1259717 (PMC10719837; doi:10.3389/fpubh.2023.1259717)
Supplement: Supplementary file 1 [file Data_Sheet_1.docx]

**Appendix**

**Table A1. Factors of Variables**

| Province | CASEID | HLG | PFA | GDP | DOR | FISV | INP | IA |
| --- | --- | --- | --- | --- | --- | --- | --- | --- |
| Anhui  Beijing  Fujian  Ganshu  Guangdong  Guangxi  Guizhou  Hainan  Hebei  Henan  Heilongjiang  Hubei  Hunan  Jiling  Jiangsu  Jiangxi  Liaoning  Neimenggu  Ningxia  Qinghai  Shandong  Sanxi  Shanxi  Shanghai  Sichuan  Tianjin  Xizang  Xinjiang  Yunnan  Zhejiang  Congqing | AH  BJ  FJ  GS  GD  GX  GZ  HAN  HEB  HEN  HLJ  HUB  HUN  JL  JS  JX  LN  NMG  NX  QH  SD  SX  SAX  SH  SC  TJ  XZ  XJ  YN  ZJ  CQ | 1  1  1  1  0  1  1  1  1  0  1  0  1  1  0  0  1  1  1  0  1  0  1  0  1  0  1  1  1  0  0 | 1  1  1  0  1  0  0  0  1  1  0  1  1  0  1  1  0  0  0  0  1  0  0  1  1  1  0  0  0  1  1 | 0  1  1  0  1  0  0  0  0  0  0  1  1  1  1  0  1  1  1  0  1  0  1  1  0  1  0  0  0  1  1 | 1  1  1  1  1  1  1  0  0  0  1  0  1  0  1  1  0  0  0  0  1  0  0  1  1  0  0  0  0  1  1 | 1  0  1  0  1  0  0  0  1  1  1  1  1  0  1  1  0  1  0  0  1  0  0  1  1  1  0  0  0  1  0 | 1  1  1  1  0  0  1  1  1  0  1  0  1  1  0  0  0  1  0  0  1  0  1  1  0  0  1  0  1  0  0 | 1  1  0  0  1  0  0  0  1  0  1  1  0  1  1  0  0  0  0  1  0  0  0  1  1  1  0  0  1  1  1 |

**Table A2. Complex Configurations**

| Paths | Coverage | Unique Coverage | Consistency |
| --- | --- | --- | --- |
| PFA*GDP*DOR*IA  ～PFA*～DOR*～FISV*～INP*～IA  ～PFA*～GDP*～DOR*～FISV*～INP PFA*～GDP*FISV*～INP*～IA  PFA*GDP*FISV*～INP*IA  PFA*～GDP*FISV*INP*IA  ～GDP*DOR*FISV*INP*IA  ～PFA*GDP*～DOR*FISV*INP*～IA  ～PFA*GDP*～DOR*～FISV*INP*IA | 0.26087  0.173913  0.130435  0.0869565  0.217391  0.0869565  0.0869565  0.0434783  0.0434783 | 0.130435  0.0869565  0.0434783  0.0869565  0.0869565  0.0434783  0.0434783  0.0434783  0.0434783 | 1  1  1  1  1  1  1  1  1 |

**Table A3. Concise Configurations**

| Paths | Coverage | Unique Coverage | Consistency |
| --- | --- | --- | --- |
| ～DOR*～INP  GDP*IA  ～PFA*FISV  FISV*～INP*～IA  ～GDP*FISV*INP | 0.347826  0.391304  0.0869565  0.0869565  0.130435 | 0.217391  0.304348  0.0434783  0.0434783  0.0869565 | 1  1  1  1  1 |

**Table A4. Intermediate Configurations**

| Paths | Coverage | Unique Coverage | Consistency |
| --- | --- | --- | --- |
| PFA*GDP*DOR*IA  ～PFA*～DOR*～FISV*～INP*～IA  ～PFA*～GDP*～DOR*～FISV*～INP PFA*～GDP*FISV*～INP*～IA  PFA*GDP*FISV*～INP*IA  PFA*～GDP*FISV*INP*IA  ～GDP*DOR*FISV*INP*IA  ～PFA*GDP*～DOR*FISV*INP*～IA  ～PFA*GDP*～DOR*～FISV*INP*IA | 0.26087  0.173913  0.130435  0.0869565  0.217391  0.0869565  0.0869565  0.0434783  0.0434783 | 0.130435  0.0869565  0.0434783  0.0869565  0.0869565  0.0434783  0.0434783  0.0434783  0.0434783 | 1  1  1  1  1  1  1  1  1 |
